# Mindfulness-Based Baduanjin Exercise for Depression and Anxiety in People with Physical or Mental Illnesses: A Systematic Review and Meta-Analysis

**DOI:** 10.3390/ijerph15020321

**Published:** 2018-02-12

**Authors:** Liye Zou, Albert Yeung, Xinfeng Quan, Stanley Sai-Chuen Hui, Xiaoyue Hu, Jessie S. M. Chan, Chaoyi Wang, Sean David Boyden, Li Sun, Huiru Wang

**Affiliations:** 1 Department of Sports Science and Physical Education, the Chinese University of Hong Kong, Shatin, Hong Kong, China; hui2162@cuhk.edu.hk (S.S.-C.H.); moonicahu@link.cuhk.edu.hk (X.H.); 2 Depression Clinical and Research Program, Massachusetts General Hospital, Harvard University, Boston, MA 02114, USA; ayeung@mgh.harvard.edu (A.Y.); SBOYDEN@partners.org (S.D.B.); 3 The South Cove Community Health Center, Boston, MA 02111, USA; 4 Department of Material Science and Engineering, Sichuan University-Pittsburgh Institute, Chengdu 610065 China; xinfeng.quan@gmail.com; 5 Department of Social Work and Social Administration, Centre on Behavioral Health, The University of Hong Kong, China; chansm5@hku.hk; 6 Department of Physical Education and Sports Science, Jilin University, Changchun 130012, China; chaoyiw@gmail.com; 7 School of Humanities and Social Science, the Chinese University of Hong Kong, Shenzhen 518172, China; sallysuncuhk@gmail.com; 8 Department of Physical Education, Shanghai Jiaotong University, Shanghai 200240, China

**Keywords:** mindfulness, Baduanjin, depression, anxiety

## Abstract

Objectives: we used a quantitative method to systematically synthesize the emerging literature and critically evaluate the effects of Baduanjin on depression and anxiety in people with physical or mental illnesses. Additionally, we determined if the number of total Baduanjin training sessions is associated with decreased anxiety and depression levels. Methods: both English and Chinese databases were searched for potential studies published between January 1982 and October 2017. The eligible randomized controlled trials were considered for meta-analysis. Effect size (Hedge’s g) was computed for the pooled effects while the random-effect model was set. For moderator analysis; Subgroup meta-analysis for categorical variables and meta-regression for continuous variables were performed. Results: the aggregated result has shown a significant benefit in favour of Baduanjin on anxiety (Hedge’s g = −0.99; CI −1.63 to −0.74) and depression (Hedge’s g = −1.07; CI −1.3 to −0.83). For continuous potential moderators; meta-regression indicated a significant effect for total hours in Baduanjin practice (*β* = −0.0053; 95% CI −0.009 to −0.0014; *p* = 0.008). With regard to depression; meta-regression indicated a significant effect for total sessions of Baduanjin practice (*β* = −0.0023; 95% CI −0.006 to −0.0004; *p* = 0.028). Conclusions: the encouraging findings indicate the efficacy of Baduanjin exercise in reducing depression and anxiety symptoms in people with physical or mental illnesses. However; the results should be interpreted with caution because of existing methodological limitations (e.g., high risk of bias; Baduanjin combined with other behavioral interventions; and heterogeneity of control groups).

## 1. Introduction

Baduanjin (also called Eight Section Brocade) is a traditional Chinese mind-body exercise routine, characterized by slow, coordinative, sequential movements [1]. Many historians believe that Baduanjin was initially created by the ancient Chinese National Hero (Yue Fei) in the Song Dynasty to help his soldiers recover from their bodily injuries and prepare for future battles [1]. With the passage of time, and particularly with the establishment of the Chinese Health Qigong Association (CHQA), movements in the Baduanjin form have been developed and reinforced to meet needs of individuals for both physical and psychological wellbeing [2]. When compared to the complex movements in Tai-Chi [3,4], one of the most popular Chinese traditional Qigong exercises, Baduanjin is easy-to-learn because it involves only eight separate movements [5]. Each individual movement needs to be practiced on both the left and right sides of the body, while integrating deep rhythmic breathing, a meditative mind, and musculoskeletal stretching and relaxation [6].

In recent years, experimental and observational studies were conducted to investigate the effects of Baduanjin on health-related parameters and disease-specific symptoms in different age groups among people with physical or mental illness. Specifically, these empirical evidences indicated that Baduanjin training could be an effective intervention program for physiological benefits (e.g., blood pressure, heart rate, and cardiorespiratory endurance) [7], attenuating bone mineral density loss [8], slowing down the process of cognitive decline (e.g., memory, attention, executive function) [9] strengthening physical function (e.g., postural stability, flexibility, leg power, and mobility) in aging populations [10,11], and alleviating chronic musculoskeletal pain [12]. After a growing body of research literature on this topic emerged, multiple meta-analytic reviews were conducted, indicating that Baduanjin could effectively alleviate symptoms in people with hypertension [5] and improve sleep quality [13], modulate blood lipid metabolism [14], and improve physical function [15].

The World Health Organization reported that an estimated 450 million people suffer from mental illness (e.g., stress, anxiety, depression, and mood disturbance) worldwide, and a quarter of these individuals experience these psychological symptoms for their entire life [16]. It is worth noting that mental illness is considered the primary risk factor for disability in people aged 15 or older [17]. Noteworthily, people who live with a chronic physical illness (e.g., older adults with cognitive impairment or poor postural stability, stroke, arthritis, asthma, heart disease, Type 2 Diabetic mellitus, and breast cancer) have a greater risk for developing anxiety and/or depression [18,19]. They not only generate substantial economic burdens in their families but also challenge national healthcare systems [20]. Based on Traditional Chinese Medicine, Baduanjin exercise involves mind-body integration practice, which may alleviate stress, anxiety, and depression. Cheng [21] recently attempted to evaluate the effects of Baduanjin on psychological health in a review study, but no definitive conclusion was made on Baduanjin for psychological well-being, particularly in people with physical or mental illnesses. Therefore, a quantitative method to systematically synthesize the emerging literature and critically evaluate the effects of Baduanjin on psychological well-being in people with physical or mental illnesses is needed. Additionally, we want to determine if the total number of sessions in Baduanjin practice is associated with decreased anxiety and depression levels. More importantly, we could provide scientific recommendation to health professionals for symptom management to improve quality of life in people with physical or mental illness.

## 2. Methods

### 2.1. Study Registration

The present study protocol was registered in the International Prospective Register of Systematic Reviews (No. CRD42017074449) for eliminating duplicates. The detailed information of this systematic review and meta-analysis is reported following the Preferred Repointing items for Systematic Reviews and Meta-Analyses (PRISMA) guidelines [22].

### 2.2. Search Strategy

Both English (Pubmed, Embase, Cochrane Library, web of science, Psycinfo, and the Who International Clinical Trials Registry Platform) and Chinese (Chinese National Knowledge Infrastructure, Wanfang, and the Chinese Clinical Trial Registry) databases were used for literature search. We searched for potential studies published between January 1982 and October 2017. To ensure inclusion of all relevant articles, the search terms used in a combined manner are as followed: “Baduanjin” or “eight section brocades” and “depression” or “anxiety” or “emotional well-being” or “mental well-being” or “stress” “psychological well-being.” Also, relevant studies were manually identified based on cross-referencing searches; reference lists of selected articles and reviews were further screened.

### 2.3. Inclusion Criteria and Study Selection

Only randomized controlled trials (RCT) were considered eligible for separate meta-analysis. Both non-randomized controlled studies (NRCT) and Pretest and posttest study (PPS) as experimental studies were included to reinforce understanding regarding the effects of Baduanjin on improving psychological outcomes. Such RCTs must include at least 20 adults diagnosed with any mental (depression, anxiety, or mood) or physical illness (e.g., fatigue, diabetic mellitus, cancer, drug addiction, heart disease, stroke, and musculoskeletal disorder). When compared to control groups (involving no intervention, educational program, drug therapy, mental therapy, or other behavioral interventions), Baduanjin as the primary component of intervention should last at least 2 weeks. Additionally, the experimental studies were only included if they reported a sum score on at least one psychological outcome. Review articles, case reports, and conference proceedings were excluded. According to the inclusion criteria, two reviewers independently filtered out irrelevant articles through reading titles and abstracts, and identified full-text articles, and removed duplicates, ultimately leading to the final number of eligible studies in this systematic review. A third party emerged to resolve disagreement between the two reviewers. The inter-rater agreement was 92.3%.

### 2.4. Quality Assessment for Each Eligible Study

Risk of bias for all selected experimental studies (RCT, NRCT, and PPS) was assessed by two independent reviewers using the 9-item Scale (because blinding of instructor and participants are not practical in non-pharmacological intervention and self-reported questionnaires for depression and anxiety were used, these three items were not considered in this systematic review) [23]. Specifically, the assessment tool involves randomization, control group, isolated Baduanjin intervention, pre-posttest design, more than 70% retention, similar baseline, intention-to-treat analysis (if missing data is present), prior sample size estimation (power analysis), and valid and reliable testing instrument relating to psychological outcomes. The maximum score of nine can be obtained for each study selected: (1) a study which scored seven or greater is considered as low risk of bias; (2) a study which scored six or lower is considered as high risk of bias.

### 2.5. Data Extraction and Data Synthesis

Two pre-created summary tables were used by two reviewers to extract data, specifically Table 1 for RCT and NRCT and Table 2 for PPS. The information in Table 1 involves the name of author, experimental location (language), initial sample size and attrition rate, participant characteristics (age and disease condition), Baduanjin intervention protocol (training period and dosage, exercise intensity, total hour and the number of total session), psychological outcome and instrument, and adverse events and follow-up assessment. In addition to the aforementioned information, Table 2 also contains the specific conclusion along with the *p* value. For both Baduanjin and control groups in the RCT, mean (M) and standard deviation (SD) of the psychological outcome and the number of participants per group at baseline and posttest were extracted. If the M and SD were not available, within-group change score from post-intervention to baseline was used. When these data were unobtainable after contacting the corresponding author of the potential studies, the individual studies were excluded.

Comprehensive Meta-Analysis Version 2.0 software (Bio. Stat. Inc., Englewood, NJ, USA) was employed to quantitatively synthesize the study findings relating the effects of Baduanjin on the outcomes of interest. Specifically, effect size (Hedges’ g) reflects the magnitude of the effects of Baduanjin on psychological outcomes, which was computed while the random-effect model was selected. The *Q*-statistic was used to determine if the heterogeneity of effect sizes across selected studies exists. If all studies shared the same effect size, the expected value of *Q* would be equal to or less than the degrees of freedom (the number of studies minus one). The I^2^ statistic (ranging from 0 to 100%, with 25% indicating low heterogeneity, 50% indicating moderate heterogeneity, and 75% indicating high heterogeneity) was computed to determine the ratio of true heterogeneity to total observed variation. For moderator analysis, Subgroup meta-analysis for categorical variables and meta-regression for continuous variables were performed. Specifically, subgroup meta-analysis involves Baduanjin intervention length (<16 weeks versus ≥16 weeks; the 8-week mind-body therapy program has been applied for mentally symptomatic management for 30 years in Massachusetts General Hospital and it is a successful mind-body intervention program. Initially, we wanted to use the 8-week Baduanjin as a cut-off, but given that study participants may miss some sessions in a Baduanjin intervention study, participants may not receive the therapeutic effect from the 8-week Baduanjin practice) [24], weekly training frequency (<5 sessions per week versus ≥5 sessions per week), session length (less than 1 h versus 1 h or longer), active (e.g., drug therapy, mental therapy, education program, and walking) and passive control (e.g., usual care, waitlist, and unaltered lifestyle) intervention, and risk of bias (low risk versus high risk). Selecting the 5-session weekly and 60-min per session was based upon the WHO recommendations on physical activity for Health (For health benefits, adults should increase their moderate intensity aerobic physical activity to 300 min per week) [25]. The number of total sessions and the total minutes of Baduanjin intervention are continuous moderator. Additionally, publication bias was evaluated using the funnel plot and Egger’s regression intercept test [26].

## 3. Results 

### 3.1. Literature Search

**Figure 1:**
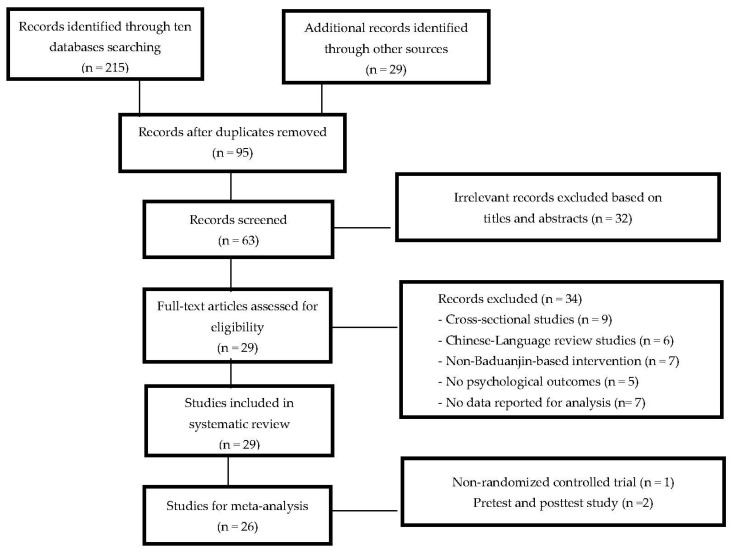
The flow of our literature search and selection process.

Figure 1 shows the flow of our literature search and selection process. After both electronic and manual searches, 244 articles were initially identified. One hundred and forty-nine were excluded because they were duplicates. Based on the titles and abstracts of the remaining articles, 32 irrelevant records were excluded. According to the inclusion criteria, 34 full-text articles were excluded because of the following reasons: (1) cross-sectional studies (*n* = 9); (2) Chinese-language review studies (*n* = 6); (3) No Baduanjin-based intervention (*n* = 7); (4) no psychological outcome (*n* = 5); (5) unobtainable data for meta-analysis (*n* = 7). Our searches resulted in 29 eligible studies, including 26 RCTs, 1 NRCT, and 2 PPS. Only RCTs were considered for meta-analysis in the present study.

### 3.2. Study Characteristics

The characteristics of the 26 RCTs and 1 NRCT selected are presented in Table 1 and two PPS in Table 2. These studies selected were published between 2010 and 2017. All experimental studies were conducted in mainland China and Hong Kong. Of the studies selected, only one study was published in English. A total sample of 2286 subjects (with an age range from 18 to 81 years old) was included in this systematic review, with a sample size of an individual study ranging from 25 to 150 participants (attrition rate ranges from 0% to 21.6%). Although all study participants suffered similar psychological symptoms (anxiety or depression), their medical/psychiatric illnesses varied greatly (chronic fatigue syndrome-like illness, perimenopausal women with depression, college students with depression, diabetic mellitus, chronic obstructive pulmonary disease, breast cancer survivors, older adults with cognitive impairment or balance impairment, glaucoma, coronary heart disease, and heroin addiction).

When compared to Baduanjin intervention group in the RCTs, the control groups received varied interventions in the selected studies, including waitlist, unaltered lifestyles, and usual care. The active control groups (without considering a combination with usual care) involved walking, physical education program, educational program, drug therapy, mental therapy, drug therapy plus education program [43], and drug therapy plus mental therapy [28,51]. The duration of Baduanjin training in the intervention groups ranged from 2 weeks to 40 weeks, and the weekly sessions ranged from 2 to 7 (each session lasted from 20 to 90 min). The number of total sessions in individual studies ranged from 16 to 200, with 18 to 200 total hours). Only three RCTs reported average heart rate (100 bpm) during Baduanjin practice [33,35,38]. Baduanjin instructor qualification was mentioned in most of the studies. Interestingly, only two psychological outcomes (depression and anxiety) were reported across all experimental studies selected. The most commonly used instruments were the Self-Rating Anxiety Scale (SAS) and Self-Rating Depression Scale (SDS), followed by Hamilton Depression Rating Scale (HAMD) and the Center for Epidemiological Studies Depression (CESD). The Hamilton Anxiety Rating Scale (HARS) and the Geriatric Depression Scale (GDS) were only used once. Only seven studies reported no adverse events during Baduanjin intervention (other studies did not mention about adverse events) [27,28,38,39,40,41,42]. Three studies used follow-up assessment (ranging from 3 to 6 months) to determine if the long-term beneficial effect of Baduanjin on psychological outcomes existed [27,28,31].

### 3.3. Methodological Quality

The inter-rater agreement about the eligible studies was 96.5%. According to the nine-item scale, a methodological quality across all experimental studies ranged from 4 to 9, with a higher score indicating lower risk of bias (Table 3). Specifically, 12 (35%) studies were considered high risk of bias, while 17 (65%) studies were rated as low risk of bias. Only one study scored 9 points [27]. Points in most of the RCTs were deducted because the Baduanjin intervention was combined with other components (e.g., education program, drug therapy, and mental therapy). For the ethical perspective, the combination of Baduanjin with other interventions is legitimate because the ultimate goal is to assist in symptomatic management in people with disease conditions. This is followed by absence of a priori sample size estimation (power analysis) and appropriate statistical method (intention-to-treat analysis while data is missed).

### 3.4. Effects of Baduanjin on Anxiety and Depression

#### 3.4.1. Baduanjin Intervention Versus Control Group on Anxiety and Depression 

**Figure 2:**
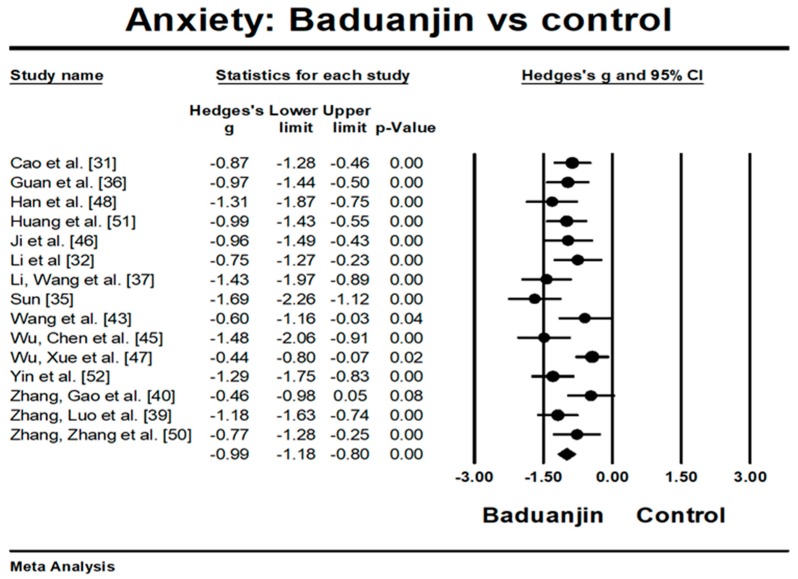
Effect of Baduanjin on anxiety.

While mean effect size was −1.03 (random-effect model), one study [49] reported large effect size (Hedge’s g = 2.63) beyond 2 SD. Additionally, Chan [27] reported a relatively small effect size (Hedge’s g = −0.47) with large sample size. The funnel plot also indicated significant asymmetry (Egger’s regression intercept = −6.698, *p* = 0.001). While these two outliers were removed for further analysis, the funnel plot of remaining studies showing no significant asymmetry (Egger’s regression intercept = −5.32, *p* = 0.063). For the meta-analysis, the 15 remaining RCTs examined the effects of Baduanjin versus control group on anxiety measured by different instruments. A higher negative value (effect size) for all anxiety-related tests indicates greater reduction of anxiety level. The aggregated result has shown a significant benefit in favour of Baduanjin on anxiety (a large effect size, but moderate heterogeneity: Hedge’s g = −0.99, *p* < 0.001, I^2^ = 55.41%) (Figure 2).

**Figure 3:**
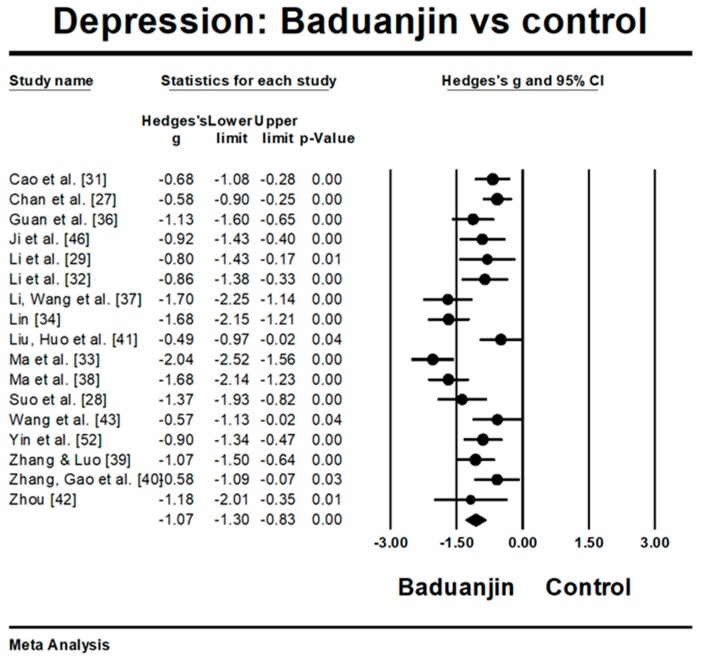
Effect of Baduanjin on depression.

Four studies reported large effect sizes beyond 2.5 SD above the mean effect size (Hedge’s g = −1.49), which are as follows: Hedge’s g = −4.79 [30], Hedge’s g = −3.12 [39], Hedge’s = −2.79 [35], and Hedge’g = −2.76 [45]. While these four studies were included for meta-analysis, the funnel plot indicated significant asymmetry (Egger’s regression intercept = −9.086, *p* = 0.009). Thus, we removed these four outliers for further analysis. The funnel plot of the remaining 17 studies showed no significant asymmetry (Egger’s regression intercept = −2.634, *p* = 0.317). For the meta-analysis, the 17 remaining RCTs examined the effects of Baduanjin versus control group on depression, measured by different instruments. A higher negative value for all depression-related tests indicates greater reduction of depression level. The aggregated result has shown a significant benefit in favour of Baduanjin on depression (a large effect size, but moderate heterogeneity: Hedge’s g = −1.07, *p* < 0.001, I^2^ = 74.24%) (Figure 3).

#### 3.4.2. Moderator Analysis

The effects of potential moderator variables were separately computed for anxiety and depression. The results of categorical and continuous moderator analysis are presented in Table 4 (anxiety) and Table 5 (depression). With regard to anxiety, there were no significant effects for type of control group (*Q*(1) = 0.113, *p* = 0.736), intervention duration (*Q*(1) = 0.55, *p* = 0.46), weekly training frequency (*Q*(1) = 1.51, *p* = 0.22), session length (*Q*(1) = 0.09, *p* = 0.76), or study quality (*Q*(1) = 0, *p* = 0.96). With regard to depression, there was a marginally insignificant effect for type of control group (*Q*(1) = 3.74, *p* = 0.053), whereas no significant effect was observed for intervention duration (*Q*(1) = 0.03, *p* = 0.87), training frequency (*Q*(1) = 2.8, *p* = 0.09), session length (*Q*(1) = 0.7, *p* = 0.4), or study quality (*Q*(1) = 0.44, *p* = 0.51).

For continuous potential moderators, meta-regression indicated a significant effect for total hours in Baduanjin practice (*β* = −0.0053, 95% CI −0.009 to −0.0014, *p* = 0.008). Specifically, a negative relationship was observed between total hours in Baduanjin practice and anxiety level. However, meta-regression indicated no significant effect for the total number of sessions in Baduanjin practice (*β* = −0.0009, 95% CI −0.005 to 0.003, *p* = 0.66) in terms of anxiety level. With regard to depression, meta-regression indicated no significant effect of total hours in Baduanjin practice (*β* = −0.0018, 95% CI −0.005 to −0.0015, *p* = 0.297), but a significant effect for total sessions in Baduanjin practice (*β* = −0.0023, 95% CI −0.006 to −0.0004, *p* = 0.028); A negative relationship was observed between total sessions and depression level.

## 4. Discussion

This systematic review and meta-analysis of randomized clinical trials on Baduanjin suggests that this traditional mind-body intervention reduces both anxiety and depression among clinically ill patients, including those with physical and mental conditions. The findings of this study add to the small, but growing literature on the effects of Baduanjin on psychological wellbeing in patients with physical or mental conditions. This is of important public health significance since anxiety and depression are highly prevalent among these patients, and Baduanjin is accessible to people of all ages and physical strength, easy to learn, and with little known side effects.

The findings in this systematic review are in accordance with the conclusions of four previously-published systematic reviews investigating the effects of mindfulness-based intervention programs (e.g., Yoga, meditation, Tai chi, and Qigong) on alleviating depression and anxiety [56,57,58,59]. Specifically, effect size estimates with respect to the effects of Baduanjin program ranged from −0.44 to −1.69 for anxiety levels and −0.49 to −2.04 for depression levels. The reduced anxiety level in this systematic review may be attributed to the features of Baduanjin which emphasize musculoskeletal relaxation, an empty state of mind, and breathing regulation. These elements in mindfulness-based Baduanjin exercise play a very important role in reducing anxiety [60,61,62]. For instance, a neurophysiological study by Yackle et al. [63] indicated that rhythmic breathing regulation in an adult animal model had a calming effect on stress-related behaviors (e.g., anxiety or panic). Regarding the reduced depression level, Chan et al. [64] gave a plausible explanation by measuring adiponectin (which has an antidepressant-like function) levels in 108 females with chronic fatigue syndrome-like illness. Reduced depression scores following a 9-week Baduanjin intervention was found to be associated with increased levels of plasma adiponectin. More interestingly, this systematic review indicates that as the total sessions increase, depression level decreases. This study finding is in line with a randomized controlled trial by Chan et al. [27] which indicated that the number of Baduanjin lessons attended and the amount of self-practice in Baduanjin was significantly correlated with depressive symptom improvement.

This study included recently-published randomized clinical studies in both English and Chinese which used Baduanjin as the intervention. Such an approach is appropriate and important since thus far, most of the studies on Baduanjin were performed in Asia and were published in the Chinese language. In doing so, the contributions of researchers on Baduanjin studies published in Chinese journals are acknowledged and the findings are more representative of the studies in this area. Other strengths of this systematic review include the use of a standardized tool to assess the quality of the studies, and formal statistical analyses to evaluate the heterogeneity of the effect sizes of the studies (*Q* Statistics), the variations in the frequency and duration of Baduanjin practice (moderator analyses), and the extent of asymmetry of effect sizes (funnel plot).

Nonetheless, these findings should be interpreted in light of the following methodological limitations. First, many of the studies included had significant flaws in their design. About one-third of the studies in this systemic review had high risk of bias. One of the most important drawbacks is that Baduanjin was offered as not as a monotherapy, but as an adjunctive treatment to the interventions received by the patients. It may be hard to tease out whether the outcomes were due to Baduanjin alone, to a combination of interventions, or to the conventional treatment received by the patients. It must be acknowledged that during the Baduanjin intervention period, people with physical or mental conditions are asked to discontinue their usual care or mental therapy/drug therapy (e.g., patients with breast cancer or moderate depression), which is impractical regardless of ethical and rehabilitation perspectives. In addition, a variety of interventions were received by control groups which made interpretations of outcomes difficult. Second, although this systematic review provided an implication that as the total hours of Baduanjin practice increases, anxiety level decreases, the duration of the Baduanjin practice varied widely among different studies. This makes it hard to make specific recommendations on how frequent and how long practices should be. Third, most of these studies examined psychological factors as secondary goals of the study, and consequently, the findings may not be applicable to patients with psychiatric disorders like major depressive disorder or generalized anxiety disorder. In addition, the questionnaires used to assess anxiety and depression differ from study to study and they may have varying levels of reliability and validity. Fourth, all studies were performed in China or Hong Kong and the participants were predominantly Chinese. It is unclear whether the results are generalizable to non-Chinese populations. Fifth, a high number of different health conditions make the samples difficult to compare. Sixth, the wide age range can also be a confounding factor if age is not controlled for in the data analysis. Lastly, positive trials are more likely to be published than those with non-significant results. Study publication bias and outcome reporting bias might have existed in the included studies, and the effect sizes of Baduanjin might have been overestimated.

## 5. Conclusion

In conclusion, the current literature suggests that Baduanjin may have positive psychological effects. Yet, many of the studies to date had significant methodological limitations which might have influenced the outcomes of this meta-analysis. More RCTs with rigorous research design are needed to establish the efficacy of Baduanjin in improving psychological well-being and its potential to be used in interventions for populations with various clinical conditions.

## Figures and Tables

**Table 1:** Summary table relating to study.

Author [Reference]	Location (Language)	ISZ (AR)	Study Participants	Intervention Protocol			Exercise Intensity	Outcome Measured	Adverse Event; Follow-Up
				Training Duration and Dosage (Qualification of Instructor)	Total Hour	No. of Session			
*Randomized controlled trials*									
Chan et al. (2014) [27]	Hong Kong, China (English)	150 (13.3%)	CFS-like illness with mild anxiety and depression (a mean age of 39)	BJ: 16 90-min sessions for 9 weeks (a Qigong master) (daily 30-min home practice); CG: Waitlist	24	16	NR	Anxiety and depression (HADS)	No; 3-month
Suo, Yu et al. (2016) [28]	Nanjin, China (Chinese)	60 (0%)	Perimenopasual women with depression (aged 44–56)	BJ: 7 × 60 min/wk for 12 weeks (trained nurse) + mental therapy (2 × 30 min/wk for 12 weeks) + usual drug therapy; CG: mental therapy (2 × 30 min/wk for 3 weeks) + usual drug therapy	84	84	NR	Depression (HAMD)	No/ 6-month
Li et al. (2014) [29]	Hengyan, China (Chinese)	45 (11.1%)	College students with depression (aged 20–23)	BJ: 5 × 60 min /wk for 40 weeks (an Qigong instructor); CG: Usual physical education classes (training volume was NR)	200	200	NR	Depression (SDS)	NR; No
Yang et al. (2017) [30]	Zhengzhou, China (Chinese)	110 (4.5%)	Type 2 DM patients with depression (aged 32–70)	BJ: 7× 60 min/wk for 12 weeks (an Qigong instructor) + usual care; CG: 2 × 30 min/wk for 3 weeks (educational program) + usual care	84	84	NR	Depression (HAMD)	NR No
Cao et al. (2016) [31]	Nangjin, China (Chinese)	103(0.97%)	COPD patients with anxiety and depression (a mean age of 70.49)	BJ: 4 × 30 min/wk for 24 weeks (trained instructors) + usual care; CG: 4 × 30 min/wk for 24 weeks (self-selected pace walking) + usual care	48	96	NR	Anxiety (SAS) Depression (SDS)	NR; 12-week
Li et al. (2017) [32]	ShanXi, China (Chinese)	68(10.3%)	Patients with breast cancer (a mean age of 45.45)	BJ: 5 sessions (session length was NR; trained nurse)/wk for 12 weeks + usual care CG: Usual care	NA	60	NR	Anxiety (SAS) Depression (SDS)	NR; No
Ma et al. (2010) [33]	Tangshan, China (Chinese)	100 (0%)	Perimenopasual women with depression (aged 45–55)	BJ: 7 × 45 min/wk for 12 weeks (instructor qualification was NR); CG: Unaltered lifestyle	63	84	AHR:100 bpm	Depression (CESD)	NR; No
Lin (2017) [34]	FuZhou, China Chinese	94(0%)	Older adults with mild cognitive impairment (aged 61–79)	BJ: 6 sessions (session length was NR; instructor qualification was NR)/wk for 24 weeks + usual care; CG: Usual care	NA	144	NR	Depression (GDS)	NR; No
Sun (2015) [35]	Jilin, China (Chinese)	65 (0%)	Type 2 DM patients (a mean age of 46.1)	BJ: 5 × 60 min/wk for 24 weeks (trained instructor) CG: Unaltered lifestyle	120	120	ARH:100 bpm	Anxiety (SAS) Depression (SDS)	NR; No
Guan et al. (2012) [36]	Hefei, China (Chinese)	80(1.25%)	Type 2 DM patients (aged 45–70)	BJ: 7 × 60 min/wk for 16 weeks + usual care CG: Usual care	112	112	NR	Anxiety (SAS) Depression (SDS)	NR; No
Li, Wang et al. (2017) [37]	Shanxi, China (Chinese)	70(4.3%)	Patients with Non-small cell lung cancer (a mean age of 56).	BJ: at least 3 × 30 min/wk for 12 weeks (trained nurse) + usual care CG: Usual care	18	36	NR	Anxiety (SAS) Depression (SDS)	NR; No
Ma et al. (2011) [38]	Tangshan, China (Chinese)	145 (0%)	Perimenopausal women with depression (aged 45–55)	BJ: 54 × 45 min/wk for 12 weeks (trained instructor) CG1: 5 × 45/wk for 12 weeks (self-selected pace walking); CG2: Unaltered lifestyle	45	60	THR: 100 bpm	Depression (CESD)	No; No
Zhang & Luo (2016) [39]	Chengdu, China (Chinese)	93(0%)	Perimenopausal women with depression and anxiety (aged 45–55)	BJ: 7 × 45 min/wk for 12 weeks (community doctor) + educational program (twice per week); CG: Twice per week (educational program)	63	84	NR	Anxiety (SAS) Depression (SDS)	No; No
Zhang, Gao et al. (2016) [40]	Bejing, China (Chinese)	60(0%)	Patients with glaucoma (aged 40–60)	BJ: 7 × 30 min/wk for 12 weeks (hospital doctor) + usual care CG: Usual care	42	84	NR	Anxiety (SAS) Depression (SDS)	No; No
Liu, Huo et al. (2012) [41]	Beijing, Ching (Chinese)	88(21.6%)	Type 2 DM patients with depression (a mean age of 64.2)	BJ: 3 × 40 min/wk for 12 weeks (trained instructor) +educational program (a total of 6 30 min educational sessions); CG: Educational program (a total of 6 30 min sessions).	24	36	NR	Depression (SDS)	No; No
Zhou (2014) [42]	Beijing, China (Chinese)	25 (0%)	Type 2 DM patients with depression (aged 51–80)	BJ: 7 × 60 min/wk for 12 weeks (NR) +usual care CG: Usual care	84	84	NR	Depression (SDS)	No; No
Wang et al. (2016) [43]	Fuzhou, China (Chinese)	50 (0%)	Patients with coronary heart disease (aged 60–70)	BJ: (Training volume was NR) for 12 weeks (NR) + usual drug therapy + educational program CG: Usual drug therapy + educational program	NA	NA	NR	Anxiety (SAS) Depression (SDS)	NR; No
Zhang, Lin et al. (2017) [44]	Liaochen, China (Chinese)	124 (0%)	Patients with depression (a mean age of 42.8).	BJ: 7 × 60 min/wk for 6 weeks (trained instructor) +usual drug therapy CG: Usual drug therapy	42	42	NR	Depression (HAMD)	NR;no
Wu, Chen et al. (2016) [45]	Changsha, China (Chinese)	60 (0%)	Patients with coronary heart disease (aged 49–79)	BJ: 5 × 60 min/wk for 12 weeks (NR) + usual care CG: Usual care	60	60	NR	Anxiety (SAS) Depression (SDS)	NR; No
Ji et al. (2012) [46]	Wuxi, China (Chinese)	62(0%)	DM patients (aged 36–81)	BJ: 7 × 45 min/wk for 8 weeks (physical therapist) + usual care + education program CG: Usual care + educational program	42	56	NR	Anxiety (SAS) Depression (SDS)	NR; No
Wu, Xue et al. (2017) [47]	Beijing, China (Chinese)	120(0%)	Older adults with anxiety and balance impaired (aged 65–80).	BJ: 2 sessions (session length was NR) per day for 30 days (hospital doctor) CG: Unaltered lifestyle	NA	60	NR	Anxiety (SAS)	NR; no
Han et al. (2017) [48]	TaiYuan, China (Chinese)	64(6.25%)	Patients with breast cancer and depression (a mean age of 46.23).	BJ: 5 × 20 min/wk for 12 weeks (five trained nurses) + usual care CG: Usual care	20	60	NR	Anxiety (SAS)	NR; No
Liu, Chen et al. (2014) [49]	Changsha, China (Chinese)	40(0%)	Type 2 DM with anxiety (a mean of 57).	BJ: 5 × 30 min/wk for 24 weeks (NR) + usual care + drug therapy CG: Usual care + drug therapy	60	120	NR	Anxiety (SAS)	NR; No
Zhang, Zhang et al. (2016) [50]	Beijing, China (Chinese)	64(3.1%)	Patients with generalized anxiety disorder (aged 22–65).	BJ: 2 × 60 min/wk for 12 weeks (NR) (daily 30 min home practice) + usual care + drug therapy CG: Usual care + drug therapy	24	24	NR	Anxiety (SAS and HARS)	NR; No
Huang et al. (2015) [51]	Zhuhai, China (Chinese)	100(12%)	Heroin addicts with anxiety (aged 18–50)	BJ: 7 × 30 min/wk for 20 weeks (NR) + mental therapy + drug therapy; CG: Mental therapy + drug therapy	70	140	NR	Anxiety (SAS)	NR; No
Yin et al. (2016) [52]	Zhuhai, China (Chinese)	88(0%)	DM patients with mental illness (a mean age of 55.47)	BJ:2 x 60 min/wk for 24 weeks (trained nurse) (daily 60min home practice) +usual care +mental therapy CG: Usual care + mental therapy	48	48	NR	Anxiety (SAS) Depression (SDS)	NR; No
*2. Non-randomized controlled studies*									
Non-randomized controlled trial									
Guan, Liu et al. (2016) [53]	Fuzhou, China (Chinese)	60 (0%)	Inpatients with depression (a mean age of 41.4)	BJ: 7 × 30 min/wk for 6 weeks (trained nurse) + usual care + drug therapy; CG1: The choreographed aerobic exercise (dosage was not reported) + usual care + drug therapy CG2: Usual care + drug therapy	21	42	NR	Depression (HAMD)	NR; No

Note: ISZ = initial sample size; AR = attribution rate; SAR = session attendance rate; BJ = Baduanjin; CG = control group; EI = exercise intensity; THR = target heart rate; AHR = average heart rate; AE = adverse event; FU = follow-up; M = month; wk = week; reps = repetitions; NA = not applicable; NR = not reported; QOI = qualification of instructor; PPS = pretest-posttest study; HADS = Hospital Anxiety and Depression Scale; HAMD = Hamilton Depression Scale; HARS = Hamilton Anxiety Rating Scale; CFS-like illness = chronic fatigue syndrome-like illness; SDS = Self-Rating Depression Scale; T2 DM = Type 2 diabetes mellitus; COPD = Chronic obstructive pulmonary disease; SAS = self-rating Anxiety Scale; SDS = self-rating Depression Scale; CESD = Center for Epidemiological Studies Depression; GDS = the Geriatric Depression Scale.

**Table 2:** Summary table for pretest and posttest studies.

Author [Reference]	Study Location	ISZ (AT)	Health Status	Duration and Dosage	Exercise Intensity	QOI	Outcome Measured	Conclusion	*p*	AE/FU
Wu, Li (2014) [54]	Beijing, China (Chinese)	68 (8.8%)	Patients with coronary heart disease and depression, with a mean age of 53.	BJ: 3 × 30 min/wk, 2 weeks	NR	Trained instructor	Depression (SDS and HAMD)	SDS: 68(11.34) vs. 67.63(11.46) HAMD: 33.23(6.9) vs. 32.60(7.13) Effectively reduced depression	0.02 0.004	NR/No
Zhou, Chen et al. (2011) [55]	Tangshan, China (Chinese)	30 (0%)	Perimenopausal women with depression, with a mean age of 48.05.	BJ: Daily morning practice (training volume was not specifically reported), 6 months	NR	Trained instructor	Depression (CESD)	CESD: 25.67(5.82) vs. 19.03(4.93) Effectively reduced depression	<0.01	NR/No

Note: ISZ = initial sample size; AT = attribution rate; QOI = qualification of instructor; CESD = Center for Epidemiological Studies Depression; HAMD = Hamilton Depression Scale; SDS = Self-Rating Depression Scale.

**Table 3:** Methodological quality for randomized controlled trials and non-randomized controlled studies.

Author [Reference]	Item 1	Item 2	Item 3	Item 4	Item 5	Item 6	Item 7	Item 8	Item 9	Score
	*Randomized controlled trials or non-randomized controlled studies*									
Chan, Li et al. (2014) [27]	Yes	Yes	Yes	Yes	Yes	Yes	Yes	Yes	Yes	9/9
Suo, Yu et al. (2016) [28]	Yes	Yes	No	Yes	Yes	Yes	Yes	No	Yes	7/9
Li, Tan et al. (2014) [29]	Yes	Yes	Yes	Yes	Yes	Yes	No	No	Yes	7/9
Yang, Huang et al. (2017) [30]	Yes	Yes	No	Yes	Yes	Yes	No	No	Yes	6/9
Cao, Guo et al. (2016) [31]	Yes	Yes	No	Yes	Yes	Yes	No	No	Yes	6/9
Li et al. (2017) [32]	Yes	Yes	No	Yes	Yes	Yes	No	No	Yes	6/9
Ma, Dou et al. (2010) [33]	Yes	Yes	Yes	Yes	Yes	Yes	Yes	No	Yes	8/9
Lin (2017) [34]	Yes	Yes	No	Yes	Yes	Yes	Yes	No	Yes	7/9
Sun (2015) [35]	Yes	Yes	No	Yes	Yes	Yes	Yes	No	Yes	7/9
Guan, Wang et al. (2012) [36]	Yes	Yes	No	Yes	Yes	Yes	No	No	Yes	6/9
Li, Wang et al. (2017) [37]	Yes	Yes	No	Yes	Yes	Yes	No	No	Yes	6/9
Ma, Dou et al. (2011) [38]	Yes	Yes	Yes	Yes	Yes	Yes	Yes	No	Yes	8/9
Zhang & Luo (2016) [39]	Yes	Yes	No	Yes	Yes	Yes	Yes	No	Yes	7/9
Zhang, Gao et al. (2016) [40]	Yes	Yes	No	Yes	Yes	Yes	Yes	No	Yes	7/9
Liu, Huo et al. (2012) [41]	Yes	Yes	No	Yes	Yes	Yes	No	No	Yes	6/9
Zhou (2014) [42]	Yes	Yes	No	Yes	Yes	Yes	Yes	No	Yes	7/9
Wang, Guan et al. (2016) [43]	Yes	Yes	No	Yes	Yes	Yes	Yes	No	Yes	7/9
Zhang, Lin et al. (2017) [44]	Yes	Yes	No	Yes	Yes	Yes	Yes	No	Yes	7/9
Wu, Chen et al. (2016) [45]	Yes	Yes	No	Yes	Yes	Yes	Yes	No	Yes	7/9
Ji, Wang et al. (2012) [46]	Yes	Yes	No	Yes	Yes	Yes	Yes	No	Yes	7/9
Wu, Xue et al. (2017) [47]	Yes	Yes	Yes	Yes	Yes	Yes	Yes	No	Yes	8/9
Han, Wang et al. (2017) [48]	Yes	Yes	No	Yes	Yes	Yes	No	No	Yes	6/9
Liu, Chen et al. (2014) [49]	Yes	Yes	No	Yes	Yes	Yes	Yes	No	Yes	7/9
Zhang, Zhang et al. (2016) [50]	Yes	Yes	No	Yes	Yes	Yes	No	No	Yes	6/9
Huang, Wu et al. (2015) [51]	Yes	Yes	No	Yes	Yes	Yes	No	No	Yes	6/9
Yin, Zhao et al. (2016) [52]	Yes	Yes	No	Yes	Yes	Yes	Yes	No	Yes	7/9
Guan, Liu et al. (2016) [53]	No	Yes	No	Yes	Yes	Yes	Yes	No	Yes	6/9
Wu, Li (2014) [54]	No	No	Yes	Yes	Yes	No	No	No	Yes	4/9
Zhou, Chen et al. (2011) [55]	No	No	Yes	Yes	Yes	No	No	No	Yes	4/9

Note: Item 1 = randomization; Item 2 = control group; Item 3 = isolated Baduanjin intervention; Item 4 = pre-posttest design; Item 5 = retention ≥ 70%; Item 6 = similar baseline; Item 7 = missing data management; Item 8 = power analysis; Item 9 = validity and reliability of measure; 1 = yes (explicitly described and present in details); 2 = no (absent, inadequately described, or unclear); NA = not applicable.

**Table 4:** Moderator analysis for Baduanjin versus control group.

Categorical Moderator	Outcome	Level	No. of Studies	Hedge’s g	95% CI	I^2^, %	Test for Between-Group Homogeneity		
							*Q*-Value	df(*Q*)	*p*-Value
Intervention duration	Anxiety	<16 weeks	9	−0.99	−1.23 to −0.75	45.87%	0.55	1	0.46
		≥16 weeks	5	−1.13	−1.39 to −0.86	38.64%			
Training frequency	Anxiety	<5 sessions/week	5	−0.93	−1.21 to −0.65	44.89%	1.51	1	0.22
		≥5 sessions/week	10	−1.20	−1.52 to −0.87	74.31%			
Session length	Anxiety	Less than 1 h	8	−1.15	−1.47 to −0.83	67.61%	0.09	1	0.76
		1 h or longer	5	−1.22	−1.54 to −0.90	47%			
Control type	Anxiety	Active	7	−0.98	−1.15 to −0.80	0%	0.113	1	0.736
		passive	8	−1.04	−1.39 to −0.70	73.19%			
Study quality	Anxiety	Low risk	8	−1	−1.33 to −0.67	72.68%	0.00	1	0.96
		High risk	7	−0.99	−1.17 to −0.81	0%			
Continuous Moderator	Level	No. of Studies	β	95% Confidence Interval		*Q*-Value		df	*p*
Total hour	Anxiety	13	−0.0053	−0.009 to −0.0014		6.9		1	0.008
Number of total sessions	Anxiety	14	−0.0009	−0.005 to 0.003		0.2		1	0.66

**Table 5:** Moderator analysis for Baduanjin versus control group.

Categorical Moderator	Outcome	Level	No. of Studies	Hedge’s g	95% CI	I^2^, %	Test for between-Group Homogeneity		
							*Q*-Value	df(*Q*)	*p*-Value
Intervention duration	Depression	<16 weeks	12	−1.08	−1.39 to −0.77	78.3%	0.03	1	0.87
		≥6 weeks	5	−1.04	−1.4 to −0.68	64.98%			
Training frequency	Depression	<5 sessions/week	5	−0.84	−1.20 to −0.48	2.822	2.8	1	0.09
		≥5 sessions/week	11	−1.22	−1.50 to −0.95				
Session length	Depression	Less than 1 h	8	−1.14	−1.54 to −0.74	82.75%	0.7	1	0.4
		1 h or longer	6	−0.94	−1.19 to −0.68	37.51%			
Control type	Depression	Active	8	−0.84	−1.02 to −0.66	16%	3.74	1	0.053
		passive	9	−1.27	−1.65 to −0.89	82.16%			
Study quality	Depression	Low risk	12	−1.11	−1.41 to −0.82	76.8%	0.44	1	0.51
		High risk	5	−0.95	−1.34 to −0.57	68.97%			
Continuous Moderator	Level	No. of Studies	β	95% Confidence Interval		*Q*-Value		df	*p*
Total hour	Depression	14	−0.0018	−0.0051 to 0.0015		1.088		1	0.297
Number of total sessions	Depression	16	−0.0023	−0.006 to −0.0004		4.85		1	0.028
